# Impact of combinatorial immunotherapies in breast cancer: a systematic review and meta-analysis

**DOI:** 10.3389/fimmu.2024.1469441

**Published:** 2024-10-16

**Authors:** Sandeep Sisodiya, Vishakha Kasherwal, Jyoti Rani, Neetu Mishra, Sandeep Kumar, Asiya Khan, Mehreen Aftab, Shagufta, Payal Singh, Ekta Gupta, Pranay Tanwar, Showket Hussain

**Affiliations:** ^1^ Cellular and Molecular Diagnostics (Molecular Biology Group), ICMR-National Institute of Cancer Prevention and Research, Noida, India; ^2^ Symbiosis School of Biological Sciences, Symbiosis International (Deemed University) (SIU), Pune, India; ^3^ Department of Zoology, Meerut College, C.C.S. University, Meerut, India; ^4^ Laboratory Oncology Unit, Dr. BRA-IRCH, All India Institute of Medical Sciences, New Delhi, India; ^5^ Depatment of Life Sciences, School of Basic Sciences and Research (SBSR), Sharda University, Greater Noida, India; ^6^ Division of Clinical Oncology, ICMR-National Institute of Cancer Prevention and Research, Noida, India

**Keywords:** Combinational therapy, immunotherapy, breast cancer, systematic review, meta-analysis

## Abstract

**Background:**

Breast cancer has the highest mortality rate among all cancers affecting females worldwide. Several new effective therapeutic strategies are being developed to minimize the number of breast cancer-related deaths and improve the quality of life of breast cancer patients. However, resistance to conventional therapies in breast cancer patients remains a challenge which could be due to several reasons, including changes in the tumor microenvironment. Attention is being diverted towards minimizing the resistance, toxicity, and improving the affordability of therapeutics for better breast cancer management. This includes personalized medicine, target-specific drug delivery systems, combinational therapies and artificial intelligence based screening and disease prediction. Nowadays, researchers and clinicians are also exploring the use of combinatorial immunotherapies in breast cancer patients, which have shown encouraging results in terms of improved survival outcomes. This study attempts to analyze the role of combinational immunotherapies in breast cancer patients, and offer insights into their effectiveness in breast cancer management.

**Methodology:**

We conducted a systematic review and meta-analysis for which we selected the randomized clinical trials (RCTs) focused on completed Phase I/II/III/IV clinical trials investigating combination immunotherapies for breast cancer. The analysis aimed to assess the efficacy of combination therapies in comparison to mono-therapies, focusing on overall survival (OS), and progression-free survival (PFS).

**Results:**

We observed that, combination immunotherapies significantly (P<0.05) improved OS as compared to single-drug therapies in the Phase I with overall Risk ratio (RR) of 16.17 (CI 2.23,117.50), Phase II with an overall RR of 19.19 (CI 11.76,31.30) and for phase III overall RR 22.27 (CI 13.60,36.37). In the case of PFS, it was significant with RR: 12.35 (CI 2.14, 71.26) in Phase I RR 6.10 (CI 4.31, 8.64) in phase II, RR 8.95 (CI 6.09, 13.16) in phase III and RR 14.82 (CI 6.49, 33.82) in Phase IV of clinical trials.

**Conclusion:**

The observed improvements in overall survival and progression-free survival suggest that combination immunotherapies could serve as a better approach to breast cancer management.

## 1 Introduction

As per Globocan 2022, among all cancers, breast cancer is one of the leading causes of death in females (1–3), due to various confounding factors, such as age, lifestyle, use of oral contraceptives, lack of physical activities, obesity, high Body Mass Index including epigenetic changes resulting into complexities, heterogenicity, and drug resistance have necessitated the use of a wide range of immunotherapeutic drugs, targeted radiation, and chemotherapies (4–7). The advent of the genomics era has significantly revolutionized the generation of cancer therapeutics. A better understanding of cancer genetics and epigenetics is crucial for the development of effective cancer prevention strategies, precision diagnostics, and therapeutic regimens (8). Targeted drug therapies, gene therapy, and cancer vaccines are available as part of cancer treatment. However, over the time, cancer cells develop resistance to these treatments or undergo genetic changes, making them less effective and increasing the risk of mortality. Finding new strategies to overcome these challenges is the need of the hour to improve cancer treatment outcomes (9, 10). To address these challenges, attempts are being made to develop new treatment approaches, such as precision medicine, personalized therapies, and combination therapy, to enhance treatment outcomes (11, 12).

**Figure 1:**
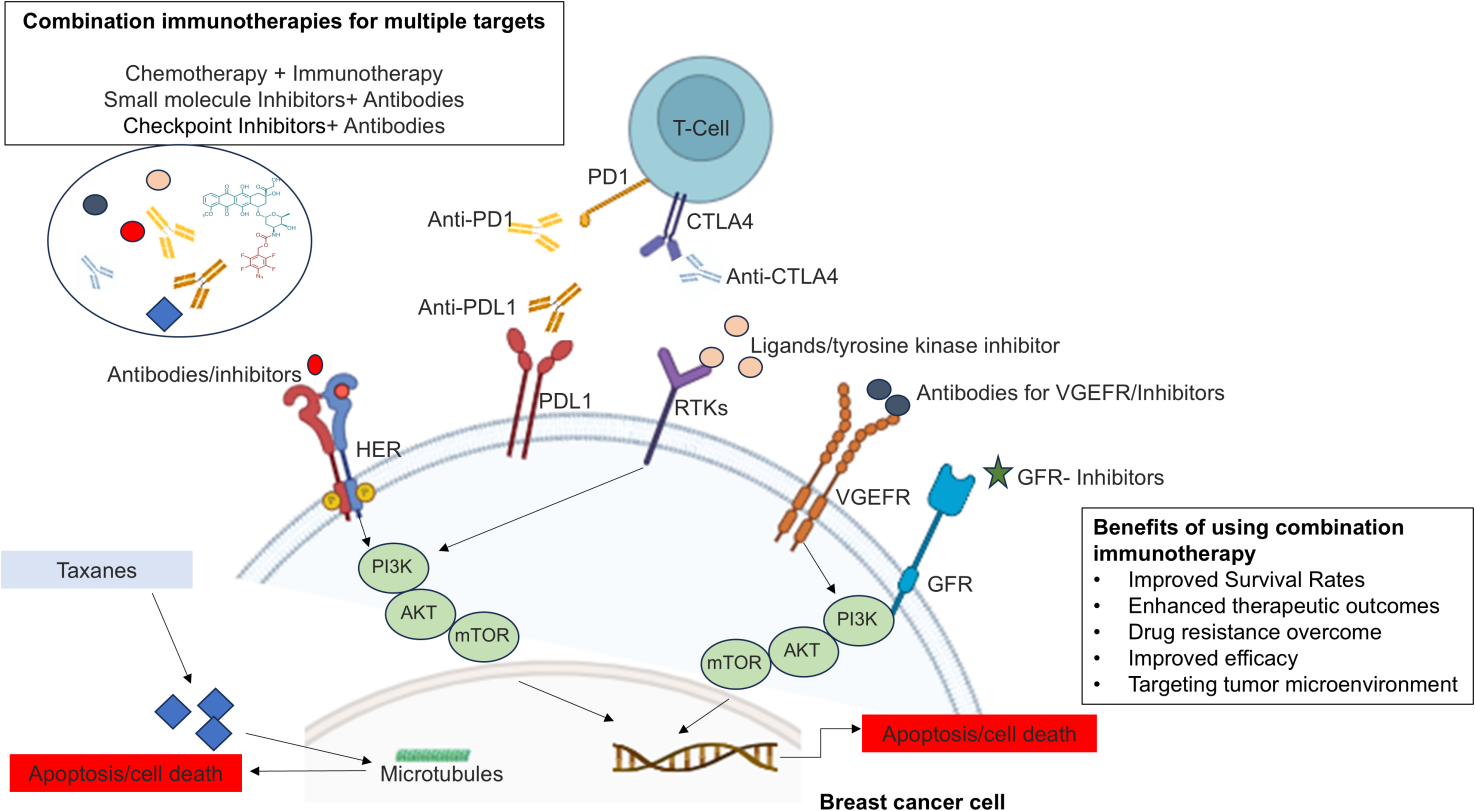
Overview of combination immunotherapy in breast cancer treatment.

Conventional therapies for treating breast cancer patients exhibit varying response rates depending upon the stages and receptor profiles of breast cancer, as well as genetic changes in cancer cells (13, 14). These reasons highlight the complexity of cancer treatment outcomes and underscore the need for personalized and tailored approaches to improve the chances of successful responses in each patient (8). Ongoing research has led to innovative combination drug therapies, such as combination immunotherapy, where more than one molecule targets different immune response pathways or different pathways to improve the effectiveness of treatment, overcome drug resistance, and reduce the likelihood of relapse. The integration of innovative therapies with existing treatments offers a potential pathway to significantly improve survival rates and reduce the overall burden of breast cancer (15, 16). The results of combination therapies have the potential to improve treatment outcomes and offer a more comprehensive approach to manage complex diseases such as breast cancer (17–20), and may reduce the mortality rate of breast cancer (**Figure 1**).

Moreover, the breast tumor microenvironment (TME) in breast cancer is a critical determinant of tumor progression, metastasis, and therapy resistance. Its complex interplay of cellular and non-cellular components creates a supportive niche for tumor growth and poses significant challenges to effective treatment. Targeting the TME, in addition to the cancer cells themselves, represents a promising strategy for overcoming resistance and improving therapeutic outcomes in breast cancer (21). Literature also suggests that combination immunotherapy offers a multifaceted approach to overcome therapy resistance in the tumor microenvironment. By targeting various components of the TME—such as immune suppression, stromal interactions, hypoxia, and antigen presentation, combination therapies can enhance the effectiveness of immunotherapy and lead to more durable responses in breast cancer. This strategy not only improves the efficacy of treatment but also addresses the underlying mechanisms of resistance, potentially leading to better clinical outcomes (22).

The emergence of personalized medicine and combination therapies has become a pivotal strategy in modern cancer treatment. Personalized medicine tailors treatment to the individual characteristics of each patient, including genetic, biomarker, and phenotypic information, allowing for more precise and effective interventions. This approach is particularly important in breast cancer, where heterogeneity among patients requires targeted therapies that addresses specific tumor profiles. The integration of personalized medicine with combination therapies enhances treatment efficacy, reduces the likelihood of resistance, and improves patient outcomes by offering a more comprehensive and tailored approach to cancer management (23, 24).

Hence, to know the effectiveness and impact of combination immunotherapy, the current systematic review and meta-analysis was focused extensively on the completed clinical trials of phases I/II/III and IV in breast cancer, where immunotherapies are used in combination. The study revealed significant outcomes in terms of overall survival (OS), and progression-free survival (PFS) in combination immunotherapies. The results of this study hold the potential to improve cancer treatment and provide insights to develop new therapies, which can ultimately improve cancer patient outcomes, especially in breast cancer. This study may also open new avenues of research in combinational immunotherapies in breast cancer with different types of stages and receptor profiles, as well as other cancers that are hard to treat due to several genetic changes and drug resistance.

## 2 Materials and methodology

### 2.1 Literature search strategy

**Figure 2:**
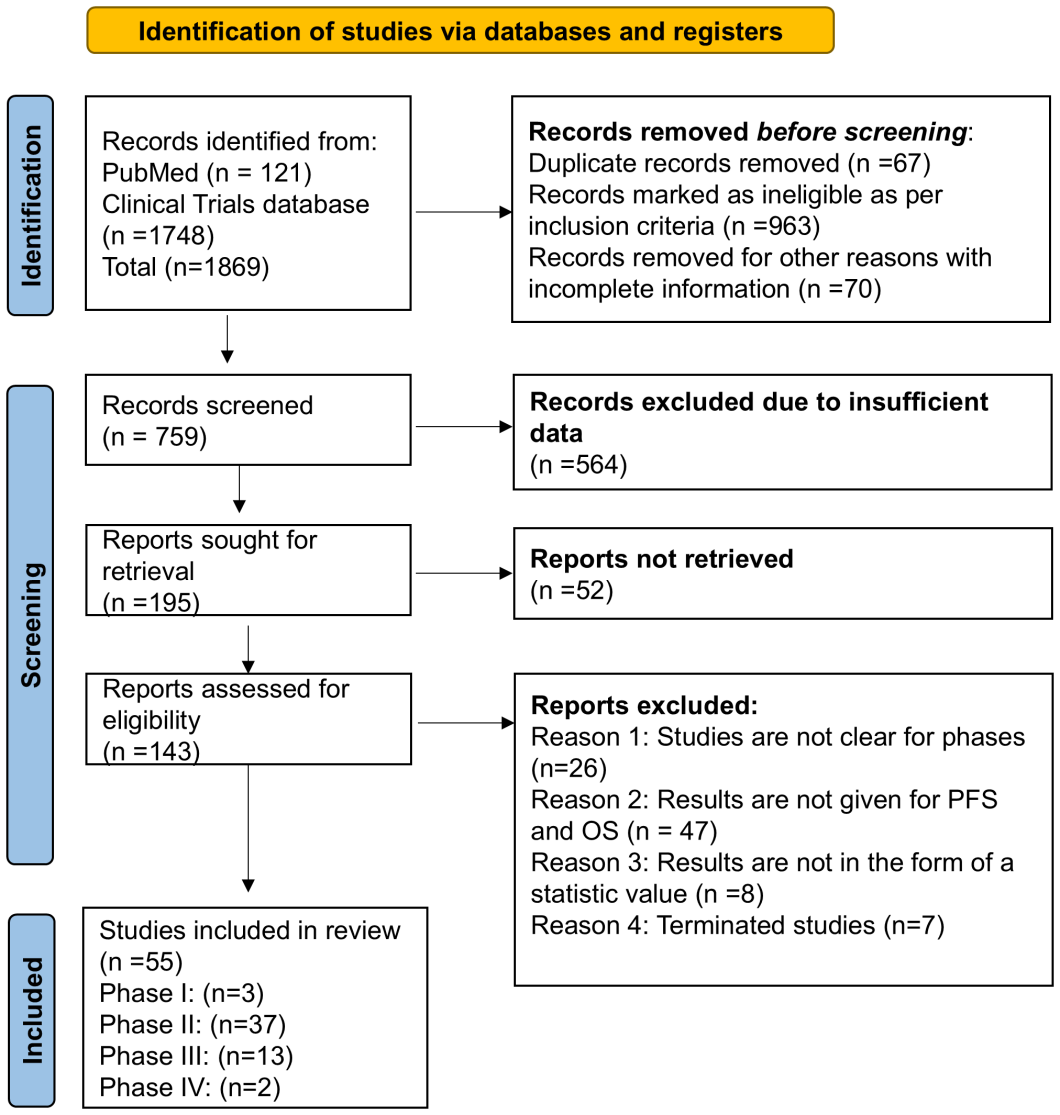
PRISMA flowchart for searching the clinical database and selection process for overall survival and progression-free survival in completed clinical trial phase I/II/III/IV in breast cancer.

A systematic review and meta-analysis study was performed as per the Preferred Reporting Items for Systematic Reviews and Meta-Analysis (PRISMA) guidelines for ensuring transparency, rigor, and consistency (**Figure 2**) (25, 26). The literature search was done through the database *“Clinicaltrials.gov.in” and PubMed* as per the PRISMA guidelines. The keywords used to identify the completed studies on *“Clinicaltrials.gov.in”* and PubMed were *“Combination therapy”, Combinational Immunotherapy” in “*breast cancer”.

The patients, intervention, comparison, outcome, and study design (PICOS) were followed to design the study.

- Patients: The studies included known breast cancer patients (females only).
- Interventions: Those studies were included that have an intervention with a drug combination with an immunotherapy drug.
- Comparators: The included studies were focused on immunotherapy compared with combination therapy (chemotherapy/radiation/inhibitors/hormonal therapy/endocrine therapy/immunotherapy + immunotherapy).
- Outcome Measures: Overall survival (OS) and progression-free survival (PFS).
- Study design: Only randomized controlled trials (RCTs) were included.

### 2.2 Data retrieval

Screening of the studies was performed by the two authors (SS & JR) on the basis of inclusion and exclusion criteria, and their results were evaluated. A final decision was made and compared with the third author’s (VK) opinion. Only those studies that have statistical analysis for overall survival (OS) and progression-free survival (PFS) in patients treated with single immunotherapy versus a combination of immunotherapy with other molecules (two or more) were selected.

#### 2.2.1 Inclusion criteria

Studies were included to compare the results of *“patients treated with one therapy versus a combination of immunotherapies with another molecule” of “randomized control clinical trials Phase I/II/III/IV” and “completed” in breast cancer*.

Additionally, only those studies that had (a) statistical median values with 95% CI intervals results of OS and PFS and (b) studies that had a combination of immunotherapies or combination of any therapy with immunotherapy were included.

#### 2.2.2 Exclusion criteria

Studies were excluded on the basis of pre-determined exclusion criteria listed below:

- Any duplicate study.
- Studies other than breast cancer.
- Results posted only for single therapy in breast cancer.
- Terminated clinical trials studies.
- Studies that did not have statistical median values and 95% CI intervals.
- Studies that did not have outcomes in the form of OS and PFS.

#### 2.2.3 Quality assessment

Quality assessment of all the included studies has been done via CONSORT questionnaire for the randomized clinical trial. All included studies hold a quality score ranging from 22 to 25, which indicates that these were of high quality for the purpose of meta-analysis (27) (**Supplementary Table 1**).

We also assessed the risk of bias for randomized controlled trials (RCTs) using the Cochrane Collaboration’s Risk of Bias (RoB) tool in Review Manager software (version 5.3) (https://community.cochrane.org/help/tools-and-software/revman-5). The evaluation covered seven key domains: random sequence generation (to identify selection bias), allocation concealment (to detect selection bias), blinding of participants and personnel (performance bias), blinding of outcome assessment (detection bias), incomplete outcome data (attrition bias), selective reporting (reporting bias), and other biases (such as funding sources). The results of this assessment are shown in **Supplementary Figure 3**.

### 2.3 Statistical analysis

The OS and PFS of patients treated with the combination of immunotherapies (with another molecule or multiple immunotherapy) versus single immunotherapy alone were investigated with the help of statistical median value with 95% confidence intervals. The statistical data of our outcome was observed and determined through overall RR and heterogeneity (I^2^ statistics) in the form of percentage value. All the statistical analysis has been carried out using RevMan 5.3 software in which p<0.05 was considered significant.

## 3 Results

### 3.1 Search criteria and study selection

The initial search focused on retrieving the studies from 2013 to 2024, where 1869 studies were identified, and on the basis of inclusion and exclusion criteria, 143 were found eligible studies. After screening and sorting of studies, 55 were selected for OS and PFS in breast cancer, where phase I-03 (OS-03 and PFS-03) phase II-34,(OS-34 and PFS found in only 28 studies) phase III-14, (OS-13 and PFS found in all 14 studies) and phase IV-02 (PFS-02 and OS was not found) studies were included in the current study (**Figure 2** and **Table 1**). Additionally, studies were excluded if the results did not have OS, PFS, and 95% confidence intervals.

**Table 1:** Details of all breast cancer randomized clinical trials for combinational immunotherapies included for the analysis (Source: Clinicaltrials.gov.in and PubMed).

S. No.	Study ID	Year	Sample size	Type of breast cancer	Drug combination	Median; 95% CI interval	Median; 95% CI interval	Outcome
Clinical trial phase I in breast cancer						PFS	OS	
**1**	NCT01975831	2022	104	Non-triple negative Breast Cancer	Durvalumab + Tremelimumab	56(27 to 223)	267 (35 to 589)	Number of Subjects with treatment-emergent Adverse Events (TEAEs) [Time Frame: Up to 36 months] Progression-free Survival [Time Frame: Up to 5 years] Overall Survival [Time Frame: Up to 5 years]
**2**	NCT00426556	2014	88	Metastatic Breast Cancer	Everolimus + Trastuzumab + Paclitaxel	5.52 (4.99 to 7.69)	18.07 (12.85 to 24.11)	PFS will be censored at the date of last adequate tumor assessment., every 8 - 9 weeks until disease progression or a new lesion is identified| OS was to be reported at extension and after 3-year follow-up. The Kaplan-Meier median was used to analyze the OS., every 3 months until death
**3**	NCT03256344 (PMID: 36863095)	2024	36	Metastatic Triple Negative Breast Cancer	Talimogene Laherparepvec + Atezolizumab	5.4 (1.0 to NA)	19.2 (1.5 to NA)	Progression-free Survival (PFS) [Time Frame: Every 12 weeks (± 28 days) up to approximately 3.5 years.] Overall Survival (OS) [Time Frame: Every 12 weeks (± 28 days) up to approximately 3.5 years.]
Clinical trial phase II in breast cancer								
**1**	NCT02513472	2022	258	Neoplasm	Eribulin Mesylate + Pembrolizumab	4.1 (2.3 to 4.4)	15.5 (12.5 to 18.7)	Objective Response Rate (ORR) [Time Frame: From date of first dose of study drug administration to date of first documentation of disease progression or death, whichever occurred first (up to 3 years 11 months)]
**2**	NCT03167619	2022	45	Triple Negative Breast Cancer	Olaparib + Durvalumab	0.11 (0.07 to 0.19)	18.27 (8.18 to NA)	Overall Survival (Olaparib in Combination With Durvalumab) [Time Frame: From date of randomization until death or last patient contact, approximately 2 years] To determine the efficacy of maintenance olaparib in combination with durvalumab following platinum based chemotherapy as assessed by overall survival (OS).
**3**	NCT00733408	2018	59	Estrogen Receptor-negative Breast Cancer|HER2-negative Breast Cancer|Progesterone Receptor-negative Breast Cancer|Recurrent Breast Cancer|Stage IV Breast Cancer|Triple-negative Breast Cancer	Paclitaxel albumin-stabilized nanoparticle formulation + Bevacizumab + Erlotinib hydrochloride	9.1(7.2 to 11.1)	18.1 (15.6 to 21.7)	Overall Survival [Time Frame: Time from date of registration to date of death due to any cause, assessed up to 8 years] Kaplan-Meier survival curves will be used. Percentage of Participants With Response [Time Frame: Up to 8 years]
**4**	NCT02657343	2022	25	HER2-positive Breast Cancer	Ribociclib + T-DM1; Ribociclib + Trastuzumab: Fulvestrant	10.4(2.7 to 19.3)	7.9 (3.4 to NA)	Maximum Tolerated Dose (Mtd) And/Or Recommended Phase2 Dose (RP2D) [Time Frame: 2 years] Clinical Benefit Rate (CBR) By RECIST [Time Frame: 2]
**5**	NCT02536339	2021	40	HER2-Positive Metastatic Breast Cancer	Pertuzumab + Trastuzumab	16.26(0.03 to 55.20)	27.17 (0.82 to 57.49)	Serum pertuzumab concentrations [Time Frame: Pre-dose and post-dose during Weeks 1, 4, 10, and 16] Serum trastuzumab concentrations [Time Frame: Pre-dose and post-dose during Weeks 1, 4, 10, and 16]
**6**	NCT02924883	2021	202	Metastatic Breast Cancer	Trastuzumab Emtansine + Placebo, Trastuzumab Emtansine + Atezolizumab	6.8 (4.0 to 11.1); 8.2 (5.8 to 10.7)	NA	Progression Free Survival (PFS) as Determined by Investigator’s Tumor Assessment Using Response Evaluation Criteria in Solid Tumors (RECIST) v1.1 [Time Frame: From Baseline until disease progression or death (up to approximately 28 months)]
**7**	NCT02536794	2022	30	Estrogen Receptor Negative|Estrogen Receptor Positive|HER2/Neu Negative|Recurrent Breast Carcinoma|Stage IV Breast Cancer	MEDI4736+ Tremelimumab	4.86(3.09 to 7.89)	11.3 (7.16 to 36.6)	Toxicity of MEDI4736 in combination with Tremelimumab [Time Frame: Up to 6 months after last treatment] Toxicity will be evaluated by the number, frequency, and severity of adverse events as defined by the NCI Common Terminology Criteria for Adverse Events or CTCAE version 4.03
**8**	NCT02648477	2024	30	Estrogen Receptor Negative|Estrogen Receptor Positive|HER2/Neu Negative|Progesterone Receptor Negative|Progesterone Receptor Positive|Stage IV Breast Cancer|Triple-Negative Breast Carcinoma	Cohort 1 (Pembrolizumab, Doxorubicin Hydrochloride) Triple Negative Breast Cancer Cohort 2 (Pembrolizumab, Anti-estrogen Therapy) HR + HER2- Breast Cancer	5.2 (4.7 to NA): 1.8 (1.6 to 2.6)	15.6 (13.3 to NA): 17.2 (9.4 to NA)	Clinical Benefit Rate [Time Frame: Up to 6 months] Overall Survival (OS) [Time Frame: Up to 3 years] Progression-free Survival (PFS) [Time Frame: Up to 3 years]
**9**	NCT01670877	2022	56	Neoplasms	Neratinib + Fulvestrant+ Trastuzumab	20 (8 to NA) 24 (15.7 to 31)		This phase II study will test cancer to see if it has a HER2 mutation and, if so, see how HER2 mutated cancer responds to treatment with neratinib.
**10**	NCT03321981	2024	105	Breast Cancer Metastatic	Zenocutuzumab + Trastuzumab + Vinorelbine + Endocrine therapy	5.59 (4.11 to 7.39) 1.45 (1.45 to 2.73)	26.41 (17.51 to NA)	A total of up to 40 patients evaluable for efficacy are included in the Cohort 2.
**11**	NCT01605396	2019	80	Neoplasms	Ridaforolimus + Dalotuzumab + Exemestane	23.29 (8.71 to 38.43)		The primary hypothesis of the study is that the triplet of ridaforolimus, dalotuzumab and exemestane will improve progression free survival (PFS) compared to ridaforolimus and exemestane.
**12**	NCT00670982	2013	29	HER2-Positive, Metastatic Breast Cancer	Ridaforolimus + Dalotuzumab + Exemestane	7.8 (3.5 to 22.0)		The purpose of this research study is to determine the effects of the combination of bevacizumab, vinorelbine, and trastuzumab on participants and their cancer.
**13**	NCT01201265	2016	40	Triple Negative Metastatic Breast Cancer	Bevacizumab+ Carboplatin+ Gemcitabine	255 (157 to 465)	475.0 (358.0 to 759.0)	This multicenter study will assess the efficacy and safety of bevacizumab in combination with gemcitabine and cisplatin as first line treatment in participants with triple negative metastatic breast cancer. Participants will receive bevacizumab at a dose of 15 mg/kg intravenously (iv) every 3 weeks, plus gemcitabine (1000 mg/m2 iv) and carboplatin (iv to an area under curve [AUC]=2) on Days 1 and 8 of each 3-week cycle. Anticipated time on study treatment is until disease progression.
**14**	NCT00004888	2014	84	Recurrent Breast Cancer|Stage IV Breast Cancer |	Pegylated liposomal doxorubicin hydrochloride + Docetaxel + Trastuzumab	10.6 (5.6 to 15.7)	31.8 (23.7 to 44.9)	Phase II trial to study the effectiveness of combination chemotherapy with or without trastuzumab in treating women who have metastatic breast cancer. Drugs used in chemotherapy use different ways to stop tumor cells from dividing so they stop growing or die. Monoclonal antibodies such as trastuzumab can locate tumor cells and either kill them or deliver tumor-killing substances to them without harming normal cells.
**15**	NCT00654836	2017	32	Recurrent or Metastatic Breast Cancer	Bevacizumab + Carboplatin + ABI-007	16 (9.80 to 22.20)	21(13.48 to 28.52)	
**16**	NCT00699491	2018	48	|Recurrent Breast Carcinoma|Stage IV Breast Cancer AJCC v6 and v7	Cixutumumab + Laboratory Biomarker Analysis + Pharmacological Study + Temsirolimus	2.0 (1.5 to 3.0)		This phase I/II trial is studying the side effects and best dose of cixutumumab when given together with temsirolimus and to see how well they work in treating patients with breast cancer that has recurred (come back) at or near the same place as the original (primary) tumor or has spread to other places in the body. Monoclonal antibodies, such as cixutumumab, can block tumor growth in different ways by targeting certain cells. Temsirolimus may stop the growth of tumor cells by blocking some of the enzymes needed for cell growth. Giving cixutumumab together with temsirolimus may be a better treatment for breast cancer.
**17**	NCT01427933	2014	141	Metastatic Breast Cancer	Ramucirumab (IMC-1121B) +: Eribulin	4.4 (3.1 to 6.7)	13.5 (10.4 to 17.9)	PURPOSE: This phase II trial is studying how well giving carboplatin and paclitaxel together with bevacizumab works in treating patients with locally recurrent or metastatic breast cancer.
**18**	NCT01234402	2019	153	Metastatic Breast Cancer	Ramucirumab DP + IMC-18F1 + Capecitabine	22.1 (12.1 to 36.1) 7.3 (6.3 to 13.0)	67.4 (41.3 to 82.6) 62.1 (41.0 to 84.0)	An open-label, multicenter, randomized, Phase 2 trial in which participant with unresectable, locally advanced or metastatic breast cancer who have been previously treated with anthracycline and taxane therapy receive ramucirumab DP or Icrucumab (IMC-18F1) administered on an every-21-day cycle (in combination with oral capecitabine therapy; capecitabine is administered twice a day on Days 1-14 of each cycle). Approximately 150 participants will be randomized in a 1:1:1 ratio to either ramucirumab DP or Icrucumab (IMC-18F1) in combination with capecitabine (Arm A and Arm B, respectively) or capecitabine monotherapy (Arm C). Randomization will be stratified by triple-negative receptor status (estrogen receptor-negative, progesterone receptor-negative, and human epidermal growth factor receptor-2 [HER2/neu]-negative) (yes/no) and receipt of prior antiangiogenic therapy.
**19**	NCT00662129	2017	50	Metastatic Breast Cancer	Bevacizumab + Gemcitabine hydrochloride + Paclitaxel albuminstabilized nanoparticle formulation	0.792 (0.647 to 0.882)	24.4 (18.2 to 29.3)	Drugs used in chemotherapy, such as gemcitabine and paclitaxel albumin-stabilized nanoparticle formulation, work in different ways to stop the growth of tumor cells, either by killing the cells or by stopping them from dividing. Monoclonal antibodies, such as bevacizumab, can block tumor growth in different ways. Some block the ability of tumor cells to grow and spread. Others find tumor cells and help kill them or carry tumor-killing substances to them. Giving combination chemotherapy together with bevacizumab may kill more tumor cells.
**20**	NCT00846027	2014	90	HER-2 negative breast cancer.	Bevacizumab + Paclitaxel + Gemcitabine	11.51 (9.01 to 17.59)	27.39 (21.86 to NA)	This single-arm study assessed the efficacy and safety of first-line treatment with Avastin (bevacizumab) in combination with taxane-based chemotherapy (paclitaxel and gemcitabine) in patients with HER-2 negative breast cancer. Patients received Avastin 10 mg/kg iv, paclitaxel 150 mg/m^2 iv, and gemcitabine 200 mg/m^2 iv on Day 1 and Day 15 of each 4-week treatment cycle until disease progression, death, or withdrawal of consent.
**21**	NCT01306942	2019	37	HER2 positive Metastatic Breast Cancer	Dasatinib + Trastuzumab + Paclitaxel	23.9 (10.3 to NA)		PURPOSE: This phase II trial is studying how well giving paclitaxel albumin-stabilized nanoparticle formulation and gemcitabine together with bevacizumab works in treating patients with metastatic breast cancer.
**22**	NCT00444587	2016	114	HER2 positive Metastatic Breast Cancer	Secondline chemotherapy + Trastuzumab[Herceptin]		717 (589 to 1057)	This 2 arm study will compare the efficacy and safety of continuation or discontinuation of Herceptin treatment in combination with 2nd line chemotherapy, in patients with HER2 positive metastatic breast cancer whose condition has progressed on 1st line chemotherapy plus Herceptin. Patients will be randomized either to continue or discontinue Herceptin treatment (6mg/kg iv infusion every 3 weeks) while receiving second-line chemotherapy of the investigator’s choice. The anticipated time on study treatment is until disease progression, and the target sample size is 100-500 individuals.
**23**	NCT00811135	2015	88	HER2-Positive Breast Cancer	Bevacizumab[Avastin] + Capecitabine[Xeloda] + Trastuzumab [Herceptin]	14.2 (10.5 to 14.9)	31.8 (26.3 to 38.2)	This single arm study will assess the efficacy and safety of Avastin in combination with Herceptin and Xeloda as first-line treatment of patients with HER2-positive locally recurrent or metastatic breast cancer. Patients will receive 3-weekly treatment cycles of Herceptin (8mg/kg iv on day 1 of first cycle, followed by 6mg/kg iv maintenance dose on day 1 of subsequent cycles), Xeloda (1000mg/m2 bid po on days 1-14 of each treatment cycle) and Avastin (15mg/kg on day 2 of first treatment cycle,and on day 1 of each subsequent cycle).The anticipated time on study treatment is until disease progression, and the target sample size is <100 individuals.
**24**	NCT02260531	2021	36	Metastatic	Cabozantinib + Trastuzumab	4.1 (2.8 to 6.2)	13.8 (8.2 to NA)	This research study is evaluating the effectiveness of the drug called cabozantinib (alone or in combination with trastuzumab) as a possible treatment for advanced breast cancer in which the cancer has spread to the brain.
**25**	NCT00193063	2014	41	HER2 positive Metastatic Breast Cancer	Trastuzumab+ Gemcitabine	4 (1.9 to 5.3)	21 (11.5 to 30.5)	Due to its remarkable activity as salvage treatment in women with metastatic breast cancer as well as the additive activity observed for gemcitabine administered in combination with trastuzumab, the clinical activity of the combination of gemcitabine administered with trastuzumab represents an exciting and ideal combination to further evaluate in Her 2 over-expressing metastatic breast cancer patients.
**26**	NCT02322814	2019	169	Metastatic Triple Negative Breast Cancer	Cobimetinib + Paclitaxel + Placebo + Atezolizumab + Nab-Paclitaxel		15.57 (14.26 to NA)	This three-cohort, multi-stage, randomized, Phase II, multicenter trial will evaluate the safety and tolerability and estimate the efficacy of cobimetinib plus paclitaxel versus placebo plus paclitaxel in Cohort I, of cobimetinib plus atezolizumab plus paclitaxel in Cohort II, and of cobimetinib plus atezolizumab plus nab-paclitaxel in Cohort III in participants with metastatic or locally advanced, triple-negative adenocarcinoma of the breast who have not received prior systemic therapy for metastatic breast cancer (MBC). Participants may continue on study treatment until the development of progressive disease (PD) or the loss of clinical benefit, unacceptable toxicity, and/or consent withdrawal. The Cohort I target sample size is 12 participants for the safety run-in stage and approximately 90 participants in the expansion stage. Each of Cohorts II and III will consist of a safety run-in stage of approximately 15 participants followed by an expansion stage of approximately 15 participants
**27**	NCT01491737	2020	258	HER2-Positive and Hormone Receptor-Positive Advanced (Metastatic or Locally Advanced) Breast Cancer	Pertuzumab + Trastuzumab + Aromatase Inhibitor + Induction Chemotherapy	20.63 (14.39 to 28.35)	60.16 (47.21 to 79.01)	This randomized, open-label, two-arm, multi-center, Phase II study will evaluate the efficacy and safety of pertuzumab in combination with trastuzumab plus an aromatase inhibitor (AI) in first-line participants with HER2-positive and hormone receptor-positive advanced breast cancer. Participants will be randomized to one of two treatment arms; Arm A (pertuzumab in combination with trastuzumab plus an AI) or Arm B (trastuzumab plus an AI). Participants may also receive induction chemotherapy (a taxane, either docetaxel or paclitaxel) at the investigator’s discretion in combination with the assigned treatment arm. The anticipated time on study treatment is until disease progression, unacceptable toxicity, withdrawal of consent, or death whichever occurs first.
**28**	NCT03025880	2023	26	HER2-negative ABC	Pembrolizumab + Gemcitabine	3.1 (2 to 4.3)	8.7 (6.5 to 11.7)	This is a multicenter phase II trial, with an initial exploratory run-in-phase, to evaluate the efficacy and safety of pembrolizumab in combination with gemcitabine in patients with HER2-negative ABC that have previously received anthracyclines and taxanes (unless clinically contraindicated). In hormone receptor positive patients, previous treatment with 2 or more lines of hormone therapy will also be required. Patients must have at least one measurable lesion that can be accurately assessed at baseline and is suitable for repeated assessment by CT, MRI or plan X-ray. Approximately 53 patients (up to a maximum of 65 patients depending on the results of the run-in-phase) will be included in this trial
**29**	NCT01565083	2016	213	HER2 positive Breast Cancer	Pertuzumab + Trastuzumab + Vinorelbine	14.3 (11.2 to 17.5) 11.5 (10.3 to 15.8)		This two-cohort, open-label, multicenter, phase 2 study will assess the safety and efficacy of pertuzumab given in combination with trastuzumab (Herceptin) and vinorelbine in first line participants with metastatic or locally advanced HER2-positive breast cancer. Participants will receive pertuzumab and trastuzumab administered sequentially as separate intravenous (IV) infusions (followed by vinorelbine) and conventional sequential administration of pertuzumab and trastuzumab in separate infusion bags, followed by vinorelbine.
**30**	NCT03121352	2023	30	Metastatic Triple Negative Breast Cancer	Carboplatin + Nab-paclitaxel + Pembrolizumab	5.8 (4.7 to 8.5)		The purpose of this study is to see how effective the combination of the two chemotherapy drugs (carboplatin and nab-paclitaxel) are when added to a third drug, pembrolizumab. Pembrolizumab is an investigational (experimental) drug that works by reinvigorating the immune system, allowing it to target and destroy cancer cells. Pembrolizumab is experimental because it is not approved by the Food and Drug Administration (FDA) for this type of breast cancer treatment.
**31**	NCT00331552	2017	30	HER2-positive Breast Cancerr|Recurrent Breast Cancer|Stage IV Breast Cancer	Pegylatedliposomal doxorubicin hydrochloride + Cyclophosphamide + Trastuzumab	0.16 (0.033 to 0.77)	0.49 (0.32 to 0.76)	Drugs used in chemotherapy, such as doxorubicin hydrochloride liposome and cyclophosphamide, work in different ways to stop the growth of tumor cells, either by killing the cells or by stopping them from dividing. Monoclonal antibodies, such as trastuzumab, can block tumor growth in different ways. Some block the ability of tumor cells to grow and spread. Others find tumor cells and help kill them or carry tumor-killing substances to them. Giving more than one drug (combination chemotherapy) together with trastuzumab may be a better way to block tumor growth.
**32**	NCT01305941	2018	32	HER-2 Positive Breast Cancer	Everolimus + Vinorelbine + Trastuzumab		1.01 (.57 to 1.78)	Purpose: This study is a single-arm, open-label phase II clinical trial testing the hypothesis that daily everolimus plus weekly vinorelbine and trastuzumab will be effective, safe, and tolerable among patients with human epidermal growth factor receptor 2 (HER2)-positive breast cancer brain metastases. Once enrolled, patients will receive everolimus PO daily in combination with weekly intravenous (IV) vinorelbine and trastuzumab. Cycles will be repeated every 3 weeks (21 days). At the time of progression, patients will come off study. Participants: Up to 35 adults over 21 with HER-2 positive breast cancer that has metastasized to the brain.
**33**	NCT02971761	2024	18	Androgen Receptor Positive|Estrogen Receptor Negative|HER2/Neu Negative|Metastatic Triple-Negative Breast Carcinoma|Progesterone Receptor Negative|Stage IV Breast Cancer AJCC v6 and v7	Enobosarm + Laboratory Biomarker Analysis + Pembrolizumab	2.6 (1.9 to 3.1)	25.5(10.4 to 30.9)	This phase II trial studies the side effects and how well pembrolizumab and enobosarm work in treating patients with androgen receptor positive triple negative breast cancer that has spread to other places in the body (metastatic). Immunotherapy with monoclonal antibodies, such as pembrolizumab, may help the body’s immune system attack the cancer, and may interfere with the ability of tumor cells to grow and spread. Androgen can cause the growth of breast cancer cells. Hormone therapy using enobosarm may fight breast cancer by blocking the use of androgen by the tumor cells. Giving pembrolizumab and enobosarm may work better than pembrolizumab alone in treating patients with androgen receptor positive triple negative breast cancer.
**34**	NCT03147287	2024	220	Metastatic Breast Cancer	Fulvestrant + Palbociclib + Avelumab	8.1(3.2 to 10.7)		This research study is studying three combinations of drugs as treatments for breast cancer. The drugs involved in this study are:Fulvestrant Fulvestrant with Palbociclib Fulvestrant with Palbociclib and Avelumab
**35**	NCT04191135	2024	462	Triple Negative Breast Neoplasms	Pembrolizumab + Olaparib + Carboplatin + Gemcitabine	5.5 (4.2 to 8.3).	25.1 (18.3 to NA).	The purpose of this study is to compare the efficacy of olaparib (MK-7339) plus pembrolizumab (MK-3475) with chemotherapy plus pembrolizumab after induction with first-line chemotherapy plus pembrolizumab in triple negative breast cancer (TNBC). The primary hypotheses are: Olaparib plus pembrolizumab is superior to chemotherapy plus pembrolizumab with respect to progression-free survival (PFS). Olaparib plus pembrolizumab is superior to chemotherapy plus pembrolizumab with respect to overall survival (OS). As of Amendment 3, study enrollment was discontinued. Participants who were receiving benefit from the study intervention could continue treatment until criteria for discontinuation are met. Participants who are on study treatment or in follow-up phase will no longer have tumor response assessments by BICR.
**36**	NCT02981303	2024	64	Advanced Melanoma|Triple-Negative Breast Cancer	Imprime PGG + Pembrolizumab	RECISTv1.1 = 2.35(1.35 to 3.98). irRECIST= 2.86(1.81 to 4.11)	16.36(11.10 to 19.22)	Objective: To determine the Overall Response Rate (ORR) to Imprime PGG + pembrolizumab in subjects with advanced melanoma or metastatic TNBC Safety: To characterize the safety of Imprime PGG + pembrolizumab given in combination Hypothesis: Restore (for melanoma) or enhance (for TNBC) sensitivity to checkpoint inhibitors (CPI) by appropriate and effective stimulation of the subject’s innate and adaptive immune systems in those subjects who have failed 1st line therapy The study will incorporate Simon’s optimal 2-stage design with sample size fixed at 12 subjects each in Stage 1 for advanced melanoma and for Triple Negative Breast Cancer (TNBC) subjects. The safety criterion of ≤ 4 (or ≤ 33%) subjects with Grade 3/4 adverse events in Cycle 1 within either tumor type must be met in order to proceed to Stage 2. The starting dose is 4 mg/kg for Imprime PGG. In the event there are a total of > 4 (or > 33%) of subjects with Grade 3/4 adverse events in Cycle 1, the dose of Imprime PGG will be reduced to 2 mg/kg, and Stage 1 will be repeated at a dose of 2 mg/kg with an additional cohort of n=12 subjects. For the dose that meets the safety criterion in Stage 1, at least 1 response in melanoma subjects and 2 responses in TNBC subjects amongst the 12 subjects within each tumor type must be observed in order to proceed to Stage 2. Stage 2 will enroll an additional 17 subjects with melanoma, and 30 subjects with TNBC. For the dose that meets the Stage 1 safety criterion, success will be declared if at least 4 amongst the total of up to 29 subjects with melanoma, and 13 amongst the total of up to 42 subjects with TNBC achieve an objective response.
**37**	NCT03051659 PMID: 32880602)	2024	90	Breast Cancer	Eribulin Mesylate + Pembrolizumab	4.1(3.5 to 6.2)	13.4 (10.4 to NA)	Progression-free survival based on the Kaplan-Meier method is defined as the duration of time from study entry to documented disease progression (PD) or death. Progression Free Survival [Time Frame: 2 years] Median Overall Survival (OS) [Time Frame: Disease assessments is performed every 3 cycles (3 weeks/cycle) for the first 18 cycles. Median follow-up 10.5 months with range 0.43-19 months.]
clinical trial phase III in breast cancer								
**1**	NCT01160211	2022	442	hormone receptor positive, HER2+ metastatic Breast Cancer	lapatinib + Trastuzumab + Aromataseinhibitor	5.6(5.4 to 8.3)	0.60 (0.35 to 1.04)	PFS of Lapatinib+Trastuzumab+AI Combination vs. Trastuzumab+AI Combination [Time Frame: approximately 5 years]
**2**	NCT00876395	2017	719	HER2-overexpressing metastatic breast cancer	Everolimus + Placebo, Trastuzumab + Paclitaxel	14.49(12.29 to 17.08)	49.97 (40.84 to NA)	Progression-free Survival (PFS) Per Investigators’ Assessment Based on Local Radiology Review - Full Population [Time Frame: date of randomization to the date of first documented tumor progression or death from any cause, whichever occurs first, reported between day of first patient randomized up to about 56 months]
**3**	NCT00545077	2014	380	HER-2 Negative Breast Cancer	Letrozole + Bevacizumab+ Fulvestrant	19.3(16.5 to 22.1)	52.1 (35.79 to 68.49)	Progression-free Survival (PFS) [Time Frame: Up to 2 years] Overall Survival (OS) [Time Frame: Up to 2 years]
**4**	NCT01250379	2015	494	metastatic Breast Cancer	Bevacizumab [Avastin] + Chemotherapy	6.3(5.5 to 7.6)	19.7 (17.6 to 21.0)	Percentage of Participants Estimated to be Surviving at Months 6, 12, 18, and 24 [Time Frame: Months 6, 12, 18, and 24] Overall survival (OS) [Time Frame: approximately 42 months] Safety: Incidence of adverse events [Time Frame: approximately 42 months]
**5**	NCT01026142	2017	452	HER-2 Positive Breast Cancer	Capecitabine + Pertuzumab + Trastuzumab	11.1(9 to 13)	37.2 (33 to 42)	Progression Free Survival (Independent Assessment) [Time Frame: Tumor assessments every 9 weeks from randomization until Week 27, then every 12 weeks thereafter, until IRF-determined PD, initiation of alternative anticancer medication, or death (up to 5.5 years).]
**6**	NCT00391092	2014	424	HER-2 Positive Breast Cancer	Bevacizumab [Avastin] + Docetaxel, Herceptin	16.5(14.1 to 19.1)	38.5 (32.1 to NA)	Progression Free Survival (PFS) [Time Frame: Every 9 weeks up to Week 36, thereafter every 12 weeks until disease progression (up to the clinical cutoff of 30 June 2011, up to 4.75 years)] Overall Survival (OS) [Time Frame: Every 9 weeks up to Week 36, thereafter every 12 weeks until disease progression (up to the clinical cutoff of 30 June 2011, up to 4.75 years)]
**7**	NCT00333775	2013	736	HER-2 Negative Breast Cancer	Docetaxel + Placebo + bevacizumab	8.7(8.2 to 9.9)	NA (15.7 to NA)	Progression-free Survival [Time Frame: Baseline to the 15 Sep 2008 cut-off date (up to 2 years, 6 months) Overall Survival [Time Frame: Baseline to the 15 Sep 2008 cut-off date (up to 2 years, 6 months)]
**8**	NCT00553358	2019	455	HER2/ErbB2 over-expressing Breast Cancer	Lapatinib + Trastuzumab + Paclitaxel		9.70 (9.60 to 9.76)	Number of Participants With Pathological Complete Response (pCR) at the Time of Surgery [Time Frame: Weeks 20 to 22] Overall Survival (OS) - Median Survival Follow-up [Time Frame: From randomization up to approximately year 10]
**9**	NCT01663727	2017	481	HER-2 Negative Metastatic Breast Cancer	Bevacizumab + Paclitaxel + Placebo	11.0 (9.5 to 12.2)	28.8 (22.8 to 32.8)	Progression Free Survival (PFS) in ITT Population [Time Frame: Baseline, every 8 weeks until documented disease progression, death or clinical cut-off (up to 117.7 weeks)] Overall Survival (OS) - ITT Population [Time Frame: From randomization till death or clinical cut-off (up to 244 weeks)]
**10**	NCT01120184	2016	1095	HER-2 PositiveMetastatic Breast Cancer	Docetaxel + Paclitaxel + Pertuzumab	14.1 (10.9 to 16.8)	53.68 (48.36 to 64.36)	Progression-Free Survival (PFS) According to IRF Assessment [Time Frame: Up to 48 months from randomization until clinical cutoff of 16-Sept-2014 (at Screening, every 9 weeks for 81 weeks, then every 12 weeks thereafter and/or up to 42 days after last dose)] Overall Survival (OS) at Clinical Cutoff [Time Frame: Up to 70 months from randomization until clinical cutoff of 15-May-2016 (every 3 months until death, loss to follow-up, withdrawal, or study termination)]
**11**	NCT02019277	2024	242	HER2-positive Metastatic Breast Cancer	Enobosarm + Laboratory Biomarker Analysis + Pembrolizumab	17.02 (12.48 to 31.18)		This open-label, multicenter, Phase IIIb study will assess the safety, tolerability and efficacy of a combination therapy of intravenous (IV) pertuzumab (Perjeta), trastuzumab (Herceptin) SC, and taxane chemotherapy (docetaxel, paclitaxel or nab-paclitaxel) as first-line therapy in participants with HER2-positive metastatic breast cancer (mBC). All participants will be treated with 3-week cycles of pertuzumab IV (840 milligrams [mg] first dose; subsequent doses of 420 mg) and trastuzumab SC (600 milligrams [mg]). The taxane treatment regimen will be determined by the investigator. Participants will continue therapy until disease progression, unacceptable toxicity, or the participant withdraws consent, whichever occurs first.
**12**	NCT02819518	2023	882	Triple Negative Breast Cancer (TNBC)	Pembrolizumab + Nab-paclitaxel + Paclitaxel + Gemcitabine + Carboplatin + Normale Saline Solution	7.5 (6.3 to 7.7)	17.2 (15.3 to 19.0).	In Part 1, the safety of pembrolizumab (MK-3475) in combination with one of three different chemotherapies will be assessed in the treatment of locally recurrent inoperable or metastatic triple negative breast cancer (TNBC), which has not been previously treated with chemotherapy. the combination of pembrolizumab and chemotherapy prolongs Progression-Free Survival (PFS) compared to placebo and chemotherapy in:all participants, participants with programmed cell death-ligand 1 (PD-L1) combined positive score (CPS) ≥1 tumors, and participants with PD-L1 CPS ≥10 tumors, and the combination of pembrolizumab and chemotherapy prolongs Overall Survival (OS) compared to placebo and chemotherapy
**13**	NCT04177108	2024	242	Triple-Negative Breast Cancer	Atezolizumab + Ipatasertib + Paclitaxel + Placebo for Atezolizumab + Placebo for Ipatasertib	7.1 (5.1 to 9.3)	15.7 (12.5 to NA)	This study evaluated the efficacy and safety of ipatasertib in combination with atezolizumab and paclitaxel in locally advanced or metastatic Triple-Negative Breast Cancer (TNBC) previously untreated in this setting.
clinical trial phase IV in breast cancer								
**1**	NCT01301729	2016	32	HER2-positive Breast Cancer	Docetaxel + Paclitaxel + Trastuzumab	9.9(6.28 to 13.63)	NA (22.64 to NA)	Progression-free survival, tumour assessments according to RECIST criteria [Time Frame: up to 4 years] Overall Response Rate [Time Frame: up to 28 months]
**2**	NCT02445586	2018	52	HER2-positive Breast Cancer	Docetaxel + Pertuzumab + Trastuzumab	23.0(13.0 to 29.0)	NA (26 to NA)	Overall Response Rate (ORR) [Time Frame: Up to 24 months after the last patient in] Progression-free survival (PFS) [Time Frame: Up to 24 months after the last patient in] Overall Survival (OS) [Time Frame: Up to 24 months after the last patient in]

### 3.2 Analysis of breast cancer in different phase clinical trials

#### 3.2.1 Overall survival

In the current meta-analysis, we have analyzed the overall survival (OS) in selected studies in phase I/II/III/IV RCTs where patients receiving a combination of immunotherapy or immunotherapy with other molecules exhibited a significant difference compared to those receiving one immunotherapy alone.

**Figure 3:**
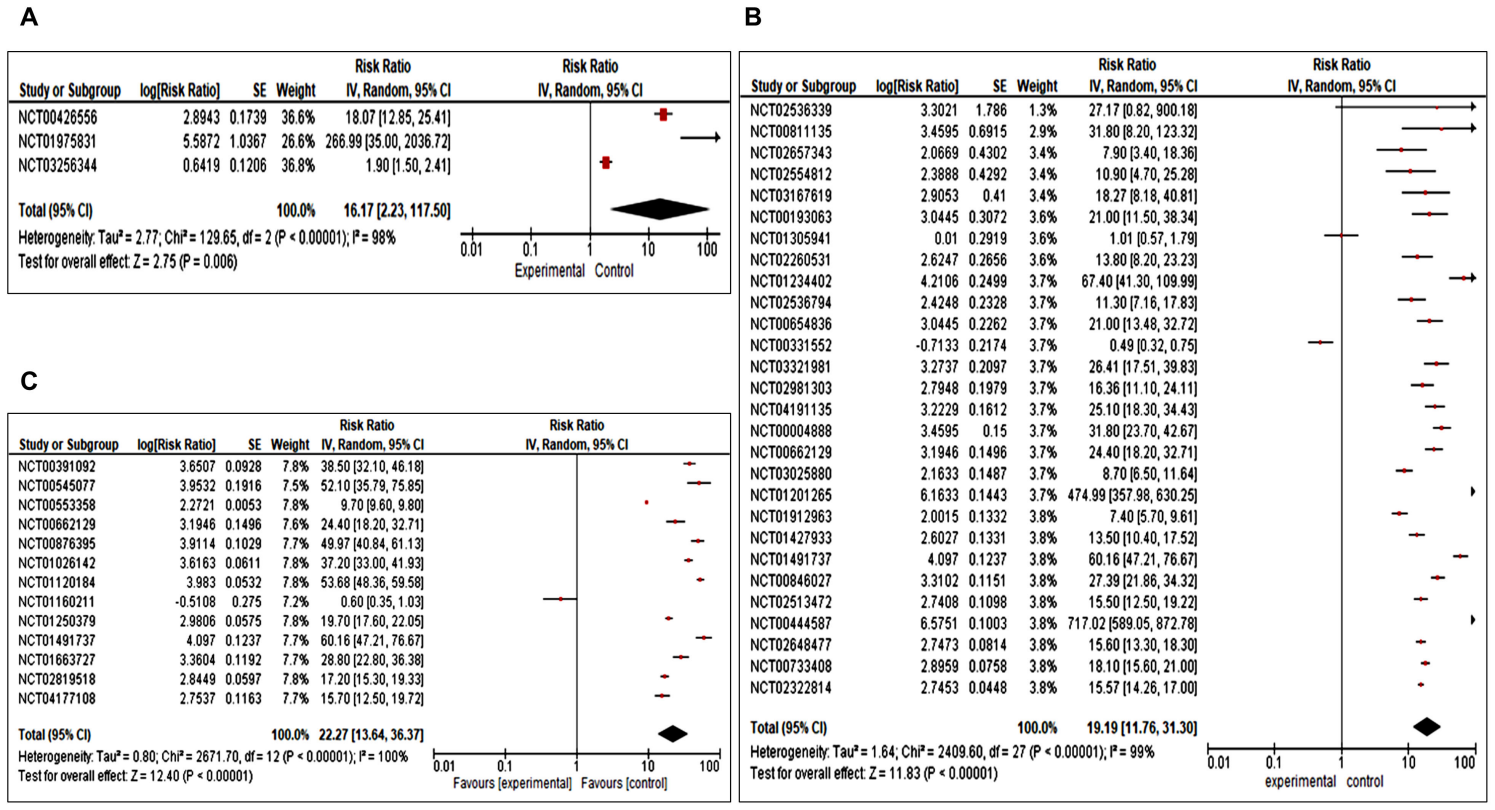
**(A–C)** Forest plot for a completed clinical trial comparing the effect of combination immunotherapies on overall survival **(A)** for phase I, **(B)** for phase II and **(C)** for phase III.

The meta-analysis revealed a high level of heterogeneity in overall survival with an overall Risk Ratio of 16.17 [(CI 2.23,117.50 (overall significance P< 0.0001)] for clinical trial phase 1, 19.19 [CI 11.76,31.30.00 (overall significance P<0.00001)] for phase II, and 22.27 [CI 13.64,36.37 (with overall significance P<0.00001)] for phase III with 95% CI interval (**Figures 3A–C**). For phase IV trials, OS data was not found in selected studies. Results of OS suggest that combination immunotherapy is highly significant in comparison to monotherapy or single immunotherapy in improving breast cancer management.

#### 3.2.2 Progression-free survival

**Figure 4:**
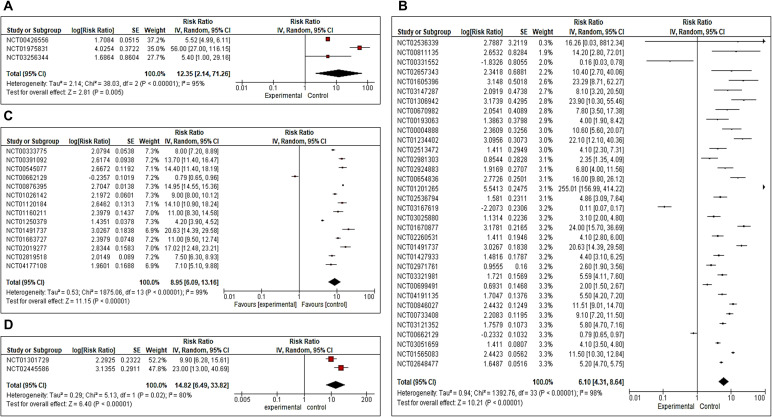
**(A–D)** Forest plot for a completed clinical trial comparing the effect of combination immunotherapies on progression-free survival **(A)** for phase II, **(B)** for phase II, **(C)** for phase III and **(D)** for Phase IV.

We also analyzed progression-free survival in all four phases I, II, III, and IV RCTs. We observed Risk Ratio of 12.35 [CI 2.14, 71.26 (overall significance P<0.0001) for phase I, 6.10 (CI 4.31, 8.64 (overall significance P<0.00001)] for phase II, 8.95 [CI 6.09, 13.16 (overall significance P<0.00001)] for phase III and 14.82 [CI 6.49, 33.82 (overall significance P<0.00001)] for phase IV (**Figures 4A–D**).

In addition, funnel plots of overall survival (**Supplementary Figures 1A–C**) and progression-free survival (**Supplementary Figures 2A–D**) were also analyzed to check the publication biases of the study. Apart from this, we have also analyzed the risk of bias through the Cochrane risk of Bias (RoB) tool in Review Manager software (version 5.3) and found a low risk of bias for eligible included studies (**Supplementary Figure 3**). Overall, the findings of the current study suggest that combination immunotherapies significantly enhance both overall survival and progression-free survival outcomes compared to single immunotherapy and better disease outcomes were observed.

## 4 Discussion

Combinatorial therapies have enabled healthcare professionals to address the limitations of traditional treatments by integrating multiple treatment modalities, such as chemotherapy, targeted therapies, immunotherapies, and radiation, in a coordinated manner for improved outcomes. Prior evidence has shown how hypo-fractioned radiotherapy was utilized in conjunction with immunotherapy to induce cancer cell death (28). Additionally, Bashraheel et al. found that combining targeted therapies like immune checkpoint inhibitors (ICIs), ligand-targeted therapeutics (LTT) or tumor-targeted superantigens (TTS) have more profound effects in treating cancer (8). Further, several other studies have also explored the effect of trastuzumab deruxtecan in solid tumors (29). Pegram et al. (1999) observed that combining trastuzumab with cisplatin led to significantly higher response rates compared to each agent when used individually. Similarly, another study explored the impact of the combination of everolimus and endocrine therapy among postmenopausal women grappling with endocrine-resistant HR+, HER2− breast cancer. This combination showed notable enhancements in progression-free survival (PFS) and objective response rates, in comparison to endocrine therapy alone (30). Moreover, meta-analysis studies have determined the efficacy of PD-1/PD-L1 inhibitors in clinical trials, highlighting their potential as effective immunotherapeutic agents across various cancer types, drug combinations, stages of treatment, and therapeutic schedules (31).

In order to evaluate the impact of combination immunotherapy vs single therapy, we performed a meta-analysis of the interventional studies with statistical data on survival outcomes in completed phase I/II/III/IV clinical trials in breast cancer. We focused on clinical trials that reported statistical interpretation of the trial in terms of Risk Ratio with 95% confidence intervals (CI) and observed that combination immunotherapies offered better overall survival (OS), and progression-free survival (PFS) outcomes to single immunotherapy. The studies were observed to be significant, with high heterogeneity in breast cancer (p<0.005) for OS and PFS. The strength of this study lies in the fact that it included only the completed phase I/II/III/IV clinical trials, providing a comprehensive assessment of the efficacy and specificity of the combination immunotherapies in breast cancer. This meta-analysis has provided us with evidence-based analysis of how combination immunotherapies are effective in overcoming the different challenges faced in cancer treatment, especially in breast cancer.

### 4.1 Limitations

Despite having 55 eligible studies for data analysis, there were limited number of studies in phase I and IV clinical trial and insufficient data for overall survival in phase IV. Additionally, data on various other survival outcome measures, such as recursion-free survival (RFS), time-to-time progression (TTP), and disease-free survival (DFS) was lacking. Further, randomized controlled trials will be necessary to validate these outcomes.

## 5 Conclusion and future prospects

Overall, our meta-analysis indicates that combinational immunotherapies involving two or more drugs or combining drugs with immune checkpoint inhibitors significantly increase overall survival (OS) and progression-free survival (PFS) in breast cancer as compared to single (one) immunotherapy. Notably, these findings provide valuable insights into the efficacy of combination immunotherapies, which can guide clinicians in making evidence-based decisions for improved breast cancer management. The future combination immunotherapies hold great potential, with numerous opportunities to enhance treatment efficacy, overcome drug resistance, and improve the quality of life in breast cancer patients particularly in complex and resistant cancer cases.

## Data Availability

The original contributions presented in the study are included in the article/**Supplementary Material**. Further inquiries can be directed to the corresponding author.
