# Supplementary material for: Impact of combinatorial immunotherapies in breast cancer: a systematic review and meta-analysis
Source: Front Immunol. 2024 Oct 16;15:1469441. doi: 10.3389/fimmu.2024.1469441 (PMC11521824; doi:10.3389/fimmu.2024.1469441)
Supplement: Supplementary file 1 [file DataSheet1.docx]

**Supplementary Material**

**Impact of combinatorial immunotherapies in breast cancer: A systematic review and meta-analysis**

Sandeep Sisodiya,^1,2^, Vishakha Kasherwal^1,2^, Jyoti Rani^1,3^, Neetu Mishra^2^, Sandeep Kumar^1^, Asiya Khan^4^, Mehreen Aftab^1^, Shagufta^1,5^, Payal Singh^1^, Ekta Gupta^6^, Pranay Tanwar^4^, Showket Hussain^1*^

^1^Cellular and Molecular Diagnostics (Molecular Biology Group), ICMR-National Institute of Cancer Prevention and Research, Noida, India

^2^Symbiosis School of Biological Sciences, Symbiosis International (Deemed University) (SIU), Pune, India

^3^Department of Zoology, Meerut College, C.C.S. University, Meerut, India

^4^Laboratory Oncology Unit, Dr. BRA-IRCH, All India Institute of Medical Sciences, Ansari Nagar, New Delhi, India

^5^Depatment of Life Sciences, School of Basic Sciences and Research (SBSR), Sharda University, Greater Noida, India

^6^Division of Clinical Oncology, ICMR-National Institute of Cancer Prevention and Research, Noida, India

***Corresponding Authors:**

**Dr. Showket Hussain**

Scientist-E

Cellular and Molecular Diagnostics (Molecular Biology Group)

ICMR - National Institute of Cancer Prevention and Research (NICPR),

Ministry of Health & Family Welfare. Govt. of India, Noida, Uttar Pradesh, India.

Email: [showket.hussain@gov.in](mailto:showket.hussain@gov.in)

**Supplementary Figures:**


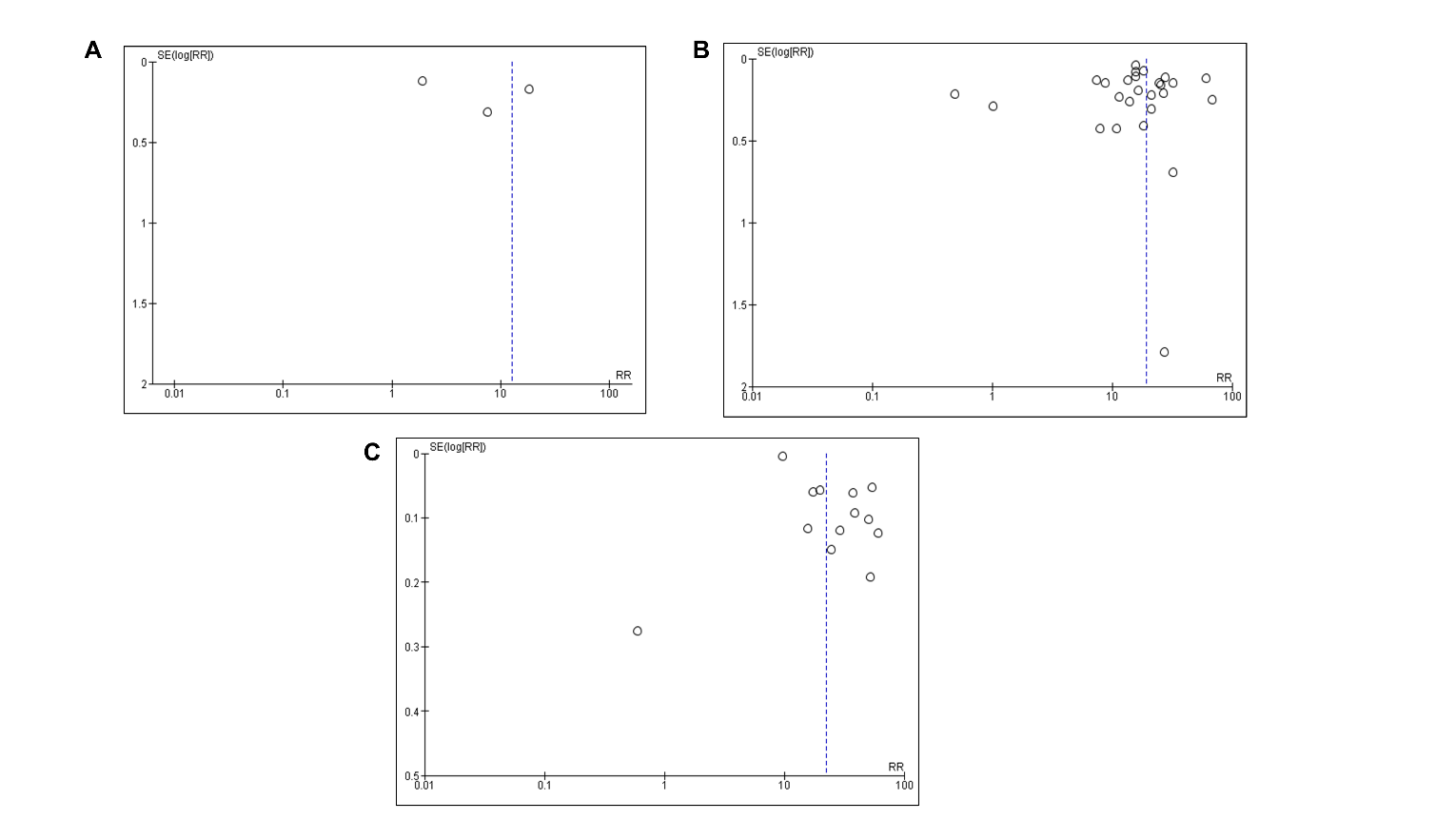


**Supplementary Figure 1 (A-C):** Funnel plot for completed clinical trial comparing the effect of combination therapies on overall survival in breast cancer (A) phase I, (B) phase II and (C) phase III.


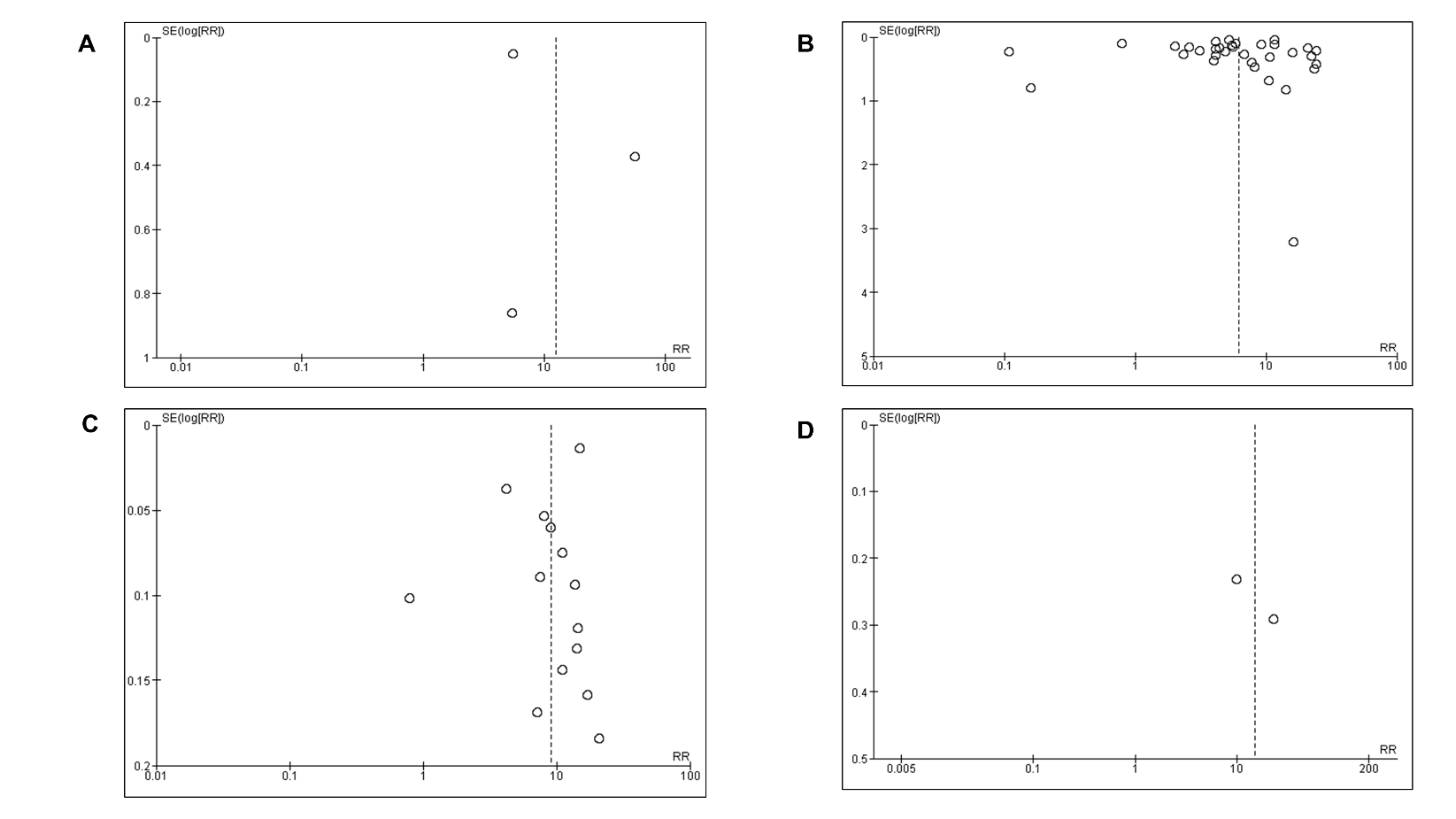


**Supplementary Figure 2 (A-D):** Funnel plot for completed clinical trial comparing the effect of combination therapies on overall survival in breast cancer (A) phase I, (B) phase II, (C) phase III and (D) phase IV

**A**

**
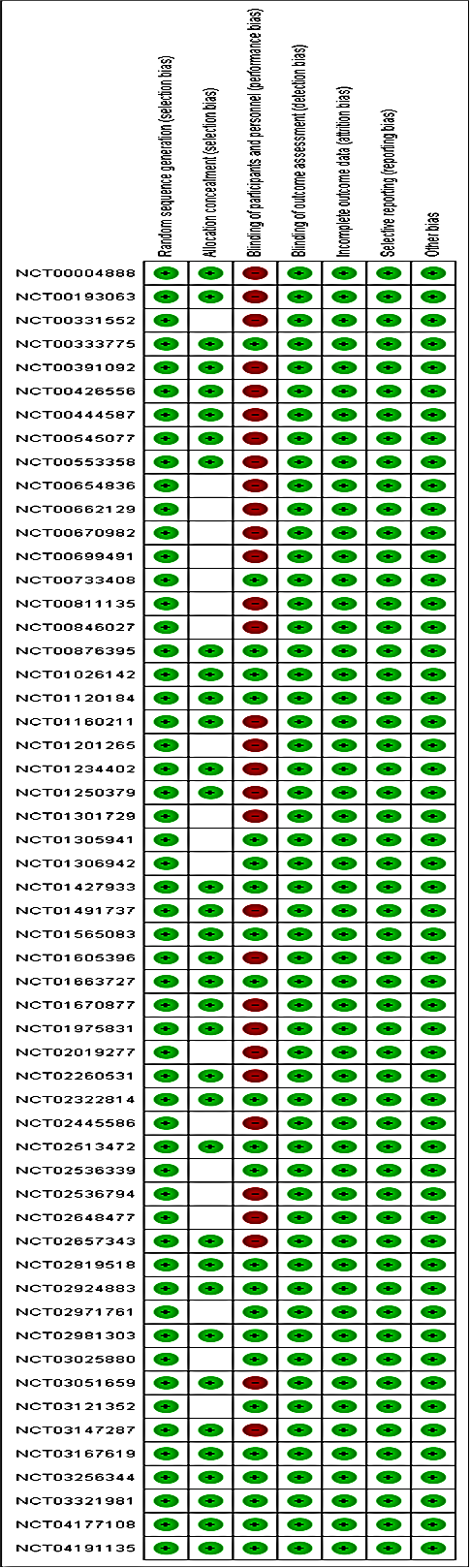
**

**
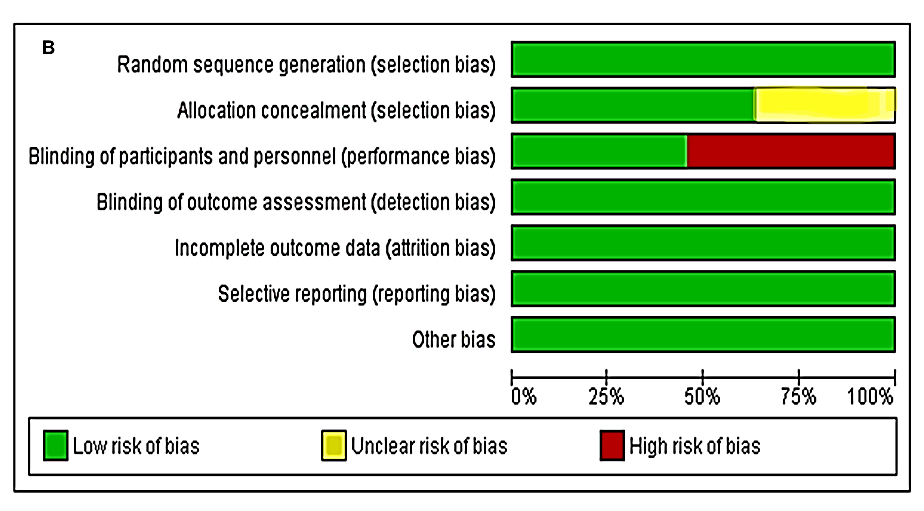
**

**Supplementary figure 3 (A &B):** Risk of bias for the included studies through Cochrane risk of Bias (RoB) tool in Review Manager software, (A) Risk of bias graph, (B) Risk of bias summary. Red stands for high risk of bias, green stands for low risk of bias and yellow/blank stands for unclear risk of bias.

**Supplementary Table 1**: Quality assessment of all included studies via CONSORT questionnaire for the randomized clinical trial

| **S.**  **No.** | **Study ID** | **Year** | **Q1** | **Q2** | **Q3** | **Q4** | **Q5** | **Q6** | **Q7** | **Q8** | **Q9** | **Q10** | **Q11** | **Q12** | **Q13** | **Q14** | **Q15** | **Q16** | **Q17** | **Q18** | **Q19** | **Q20** | **Q21** | **Q22** | **Q23** | **Q24** | **Q25** | **Score** |
| --- | --- | --- | --- | --- | --- | --- | --- | --- | --- | --- | --- | --- | --- | --- | --- | --- | --- | --- | --- | --- | --- | --- | --- | --- | --- | --- | --- | --- |
| **Clinical trial phase I in breast cancer** | | | | | | | | | | | | | | | | | | | | | | | | | | | | |
| 1 | NCT01975831 | 2022 | * | * | * | * | * | * |  | * |  |  | * | * |  | * | * | * | * | * | * |  | * | * | * | * | * | 20 |
| 2 | NCT00426556 | 2015 | * | * | * | * | * | * |  |  |  |  | * | * | * | * | * | * | * | * | * |  | * | * | * | * | * | 20 |
| 3 | NCT03256344 | 2024 | * | * | * | * | * | * |  |  |  |  | * | * | * | * | * | * | * | * | * |  | * | * | * | * | * | 20 |
| **Clinical trial phase II in breast cancer** | | | | | | | | | | | | | | | | | | | | | | | | | | | | |
| 1 | NCT02513472 | 2022 |  | * | * | * | * | * |  | * |  |  | * | * | * | * | * | * | * | * | * |  | * | * | * | * | * | 20 |
| 2 | NCT03167619 | 2022 | * | * | * | * | * | * |  | * |  |  | * | * |  |  | * | * | * | * | * |  | * | * | * | * | * | 19 |
| 3 | NCT00733408 | 2018 | * | * | * | * | * | * |  | * |  |  | * | * |  |  | * | * | * | * | * |  | * | * | * | * | * | 19 |
| 4 | NCT02657343 | 2022 | * | * | * | * | * | * |  | * |  |  | * | * | * |  | * | * | * | * | * |  | * | * | * | * | * | 20 |
| 5 | NCT02536339 | 2021 | * | * | * | * | * | * |  | * |  |  | * | * |  |  | * | * | * | * | * |  | * | * | * | * | * | 19 |
| 6 | NCT02924883 | 2021 | * | * | * | * | * | * |  | * |  |  | * | * | * |  | * | * | * | * | * |  | * | * | * | * | * | 20 |
| 7 | NCT02536794 | 2022 | * | * | * | * | * | * |  | * |  |  | * | * |  | * | * | * | * | * | * | * | * | * | * | * | * | 21 |
| 8 | NCT02648477 | 2024 | * | * | * | * | * | * |  | * |  |  | * | * | * |  | * | * | * | * | * |  | * | * | * | * | * | 20 |
| 9 | NCT01670877 | 2022 | * | * | * | * | * | * |  |  |  |  | * | * | * | * | * | * | * | * | * |  | * | * | * | * | * | 20 |
| 10 | NCT03321981 | 2024 | * | * | * | * | * | * |  | * |  |  | * | * | * | * | * | * | * | * | * |  | * | * | * | * | * | 21 |
| 11 | NCT01605396 | 2019 | * | * | * | * | * | * |  | * |  |  | * | * | * | * | * | * | * | * | * |  | * | * | * | * | * | 21 |
| 12 | NCT00670982 | 2013 | * | * | * | * | * | * |  |  |  |  | * | * | * | * | * | * | * | * | * |  | * | * | * | * | * | 20 |
| 13 | NCT01201265 | 2016 | * |  | * | * | * | * |  |  |  |  | * | * | * | * | * | * | * | * | * |  | * | * | * | * | * | 19 |
| 14 | NCT00004888 | 2014 | * | * | * | * | * | * |  |  |  |  | * | * | * | * | * | * | * | * | * |  | * | * | * | * | * | 20 |
| 15 | NCT00654836 | 2017 | * | * | * | * | * | * |  |  |  |  | * | * | * | * | * | * | * | * | * |  | * | * | * | * | * | 20 |
| 16 | NCT00699491 | 2018 | * | * | * | * | * | * |  |  |  |  | * | * | * | * | * | * | * | * | * |  | * | * | * | * | * | 20 |
| 17 | NCT01427933 | 2017 | * | * | * | * | * | * |  | * |  |  | * | * | * | * | * | * | * | * | * |  | * | * | * | * | * | 21 |
| 18 | NCT01234402 | 2019 | * | * | * | * | * | * |  | * |  |  | * | * | * | * | * | * | * | * | * |  | * | * | * | * | * | 21 |
| 19 | NCT00662129 | 2017 | * | * | * | * | * | * |  |  |  |  | * | * | * | * | * | * | * | * | * |  | * | * | * | * | * | 20 |
| 20 | NCT00846027 | 2014 | * | * | * | * | * | * |  |  |  |  | * | * | * | * | * | * | * | * | * |  | * | * | * | * | * | 20 |
| 21 | NCT01306942 | 2023 | * | * | * | * | * | * |  |  |  |  | * | * | * | * | * | * | * | * | * |  | * | * | * | * | * | 20 |
| 22 | NCT00444587 | 2016 | * | * | * | * | * | * |  | * |  |  | * | * | * | * | * | * | * | * | * |  | * | * | * | * | * | 21 |
| 23 | NCT00811135 | 2016 | * | * | * | * | * | * |  |  |  |  | * | * | * | * | * | * | * | * | * |  | * | * | * | * | * | 20 |
| 24 | NCT02260531 | 2021 | * | * | * | * | * | * |  |  |  |  | * | * | * | * | * | * | * | * | * |  | * | * | * | * | * | 20 |
| 25 | NCT00193063 | 2014 | * | * | * | * | * | * |  |  |  |  | * | * | * | * | * | * | * | * | * |  | * | * | * | * | * | 20 |
| 26 | NCT02322814 | 2023 | * | * | * | * | * | * |  | * |  |  | * | * | * | * | * | * | * | * | * |  | * | * | * | * | * | 21 |
| 27 | NCT01491737 | 2020 | * | * | * | * | * | * | * | * |  |  | * | * | * | * | * | * | * | * | * | * | * | * | * | * | * | 23 |
| 28 | NCT03025880 | 2023 | * | * | * | * | * | * |  |  |  |  | * | * | * | * | * | * | * | * | * |  | * | * | * | * | * | 20 |
| 29 | NCT01565083 | 2016 | * | * | * | * | * | * |  | * |  |  | * | * | * | * | * | * | * | * | * |  | * | * | * | * | * | 21 |
| 30 | NCT03121352 | 2023 | * | * | * | * | * | * |  |  |  |  | * | * | * | * | * | * | * | * | * |  | * | * | * | * | * | 20 |
| 31 | NCT00331552 | 2017 | * | * | * | * | * | * |  |  |  |  | * | * | * | * | * | * | * | * | * |  | * | * | * | * | * | 20 |
| 32 | NCT01305941 | 2018 | * | * | * | * | * | * |  |  |  |  | * | * | * | * | * | * | * | * | * |  | * | * | * | * | * | 20 |
| 33 | NCT02971761 | 2024 | * | * | * | * | * | * |  |  |  |  | * | * | * | * | * | * | * | * | * | * | * | * | * | * | * | 21 |
| 34 | NCT03147287 | 2024 | * | * | * | * | * | * |  | * |  |  | * | * | * | * | * | * | * | * | * |  | * | * | * | * | * | 21 |
| 35 | NCT04191135 | 2024 | * | * | * | * | * | * |  | * |  |  | * | * | * | * | * | * | * | * | * |  | * | * | * | * | * | 21 |
| 36 | NCT02981303 | 2024 | * | * | * | * | * | * |  | * |  |  | * | * | * | * | * | * | * | * | * |  | * | * | * | * | * | 21 |
| 37 | NCT03051659 | 2024 | * | * | * | * | * | * |  | * |  |  | * | * | * | * | * | * | * | * | * |  | * | * | * | * | * | 21 |
| **Clinical trial phase III in breast cancer** | | | | | | | | | | | | | | | | | | | | | | | | | | | | |
| 1 | NCT01160211 | 2022 | * | * | * | * | * | * |  | * |  |  | * | * | * | * | * | * | * | * | * |  | * | * | * | * | * | 21 |
| 2 | NCT00876395 | 2017 | * | * | * | * | * | * |  | * |  |  | * | * | * | * | * | * | * | * | * | * | * |  | * | * | * | 21 |
| 3 | NCT00545077 | 2014 | * | * | * | * | * | * |  | * |  |  | * | * | * |  | * | * | * | * | * |  | * | * | * | * | * | 20 |
| 4 | NCT01250379 | 2015 | * | * | * | * | * | * |  | * |  |  | * | * | * | * | * | * | * | * | * |  | * | * | * | * | * | 21 |
| 5 | NCT01026142 | 2017 | * | * | * | * | * | * |  | * |  |  | * | * | * |  | * | * | * | * | * |  | * | * | * | * | * | 20 |
| 6 | NCT00391092 | 2014 | * | * | * | * | * | * |  | * |  |  | * | * | * |  | * | * | * | * | * |  | * | * | * | * | * | 20 |
| 7 | NCT00333775 | 2013 | * | * | * | * | * | * |  | * |  |  | * | * |  | * | * | * | * | * | * |  | * |  | * | * | * | 19 |
| 8 | NCT00553358 | 2019 | * | * | * | * | * | * |  | * |  |  | * | * | * |  | * | * | * | * | * |  | * | * | * | * | * | 20 |
| 9 | NCT01663727 | 2017 | * | * | * | * | * | * |  | * |  |  | * | * | * |  | * | * | * | * | * |  | * | * | * | * | * | 20 |
| 10 | NCT01120184 | 2016 | * | * | * | * | * | * |  | * |  |  | * | * | * |  | * | * | * | * | * |  | * | * | * | * | * | 20 |
| 11 | NCT02019277 | 2018 | * | * | * | * | * | * |  |  |  |  | * | * | * | * | * | * | * | * | * |  | * | * | * | * | * | 20 |
| 12 | NCT02819518 | 2023 | * | * | * | * | * | * |  |  |  |  | * | * | * | * | * | * | * | * | * |  | * | * | * | * | * | 20 |
| 13 | NCT04177108 | 2024 | * | * | * | * | * | * | * | * |  |  | * | * | * | * | * | * | * | * | * |  | * | * | * | * | * | 22 |
| **Clinical trial phase IV in breast cancer** | | | | | | | | | | | | | | | | | | | | | | | | | | | | |
| 1 | NCT01301729 | 2014 | * | * | * | * | * | * |  | * |  |  | * | * |  |  | * | * | * | * | * |  | * | * | * | * | * | 19 |
| 2 | NCT02445586 | 2018 | * | * | * | * | * | * |  | * |  |  | * |  | * | * | * | * | * | * | * |  | * | * | * | * | * | 20 |
